# Influence of Nickel on Microstructure and Mechanical Properties in Medium-Carbon Spring Steel

**DOI:** 10.3390/ma17102423

**Published:** 2024-05-17

**Authors:** Qian Yu, Yuliang Zhao, Feiyu Zhao

**Affiliations:** 1 The State Key Laboratory of Rolling and Automation, Northeastern University, Shenyang 110189, China; yuquan0@126.com; 2 Materalia Group, Department of Physical Metallurgy, Centro Nacional de Investigaciones Metalurgicas (CENIM-CSIC), Av. Gregorio del Amo 8, 28040 Madrid, Spain; zhaoyl@dgut.edu.cn; 3 Neutron Scattering Technical Engineering Research Centre, School of Mechanical Engineering, Dongguan University of Technology, Dongguan 523808, China

**Keywords:** spring steel, martensite, yield strength, impact toughness, nickel content

## Abstract

The effects of adding nickel on the phase transition temperature, microstructure, and mechanical properties of medium-carbon spring steel have been investigated. The results show that adding nickel reduces the martensite start (*M*_s_) temperature, improves hardenability, and refines the sub-microstructure of the martensite, thereby improving yield stress. The yield strength of martensitic steel increases by approximately 100 MPa due to a synergistic combination of grain refinement strengthening and dislocation strengthening, with an increase in the nickel content from 0 wt.% to 1 wt.%. The cryogenic impact toughness of martensitic steel also improved with a higher nickel content due to packet and block refinement and an increase in the proportion of high-angle grain boundaries (HAGBs).

## 1. Introduction

The demand for weight reduction and an improvement in the performance of the automotive industry is increasing due to energy shortages and growing restrictions on vehicle emissions [1]. Reducing the weight of springs is of great importance in reducing fuel consumption, and one way to achieve this is by developing high-strength spring steels. However, increasing strength while maintaining other properties, such as ductility and toughness, poses a significant challenge for the development of ultrahigh-strength spring steel [2].

Over the past few decades, several approaches have been employed to enhance the strength, ductility, and toughness of spring steel. These include controlling the design of the alloy composition [3,4,5], implementing effective heat treatment [2,6,7,8,9,10], and utilizing microalloying techniques [11,12]. Medium-carbon spring steel with quenched and tempered lath martensite structures has been widely used as spring steel due to its exceptional strength, ductility, and lower decarburization sensitivity [2,4,7]. Wang et al. [4] demonstrated in their study that 38Si7 spring steel, which has a low microalloy content, exhibited an impressive performance of 20.2 GPa·% after austenitization, quenching, and subsequent medium-temperature tempering. The strength and toughness of spring steel have recently garnered attention due to the complex working conditions [3]. According to Wang et al. [5], the Ni-bearing medium-carbon spring steel achieved a good combination of 2000 MPa ultimate tensile strength (UTS), 11.1% elongation, and 38 J Charpy impact energy.

Ni and Fe are both 3D-transition metals, and Ni is a substitutional solute element, with extensive solid solubility in steel [13,14]. The addition of Ni promotes the cross-sliding of dislocations in martensitic steel [3], which reduces the tough–brittle transition temperature and increases cryogenic impact toughness by promoting deformation rather than cleavage fracture [15,16]. The addition of the Ni element can refine the microstructure and reduce the size of martensite packets and blocks, thereby increasing strength and toughness [17,18]. A further benefit of adding nickel is inhibiting the precipitation of cementite during the low temperature tempering process and improving the mechanical properties of the steels [16]. Low-temperature tempering of Ni-bearing medium carbon spring steel can provide a good combination of strength and ductility. In the study by Firrao et al. [10], the precipitation of cementite in 39NiCrMo3 can be effectively inhibited by tempering at temperatures below 240 °C, resulting in exceptional mechanical properties. Ultimately, an ultra-high tensile strength of 1800 MPa and a yield strength of 1600 MPa were achieved. Due to the challenges associated with enhancing the strength of traditional spring steel while maintaining sufficient plasticity and toughness, the spring steel in this study was designed with reference to the composition and processing of medium carbon spring steel and press hardening steel (PHS) ultrahigh-strength martensitic steel to develop a spring steel of 1900 MPa grade with high plasticity and cryogenic impact toughness.

The purpose of the present work is to investigate the influence of adding nickel (from 0 to 1 wt.%) on the phase-transition temperature, microstructural evolution, strengthening mechanisms, and impact toughness in medium-carbon spring steel.

## 2. Materials and Methods

### 2.1. Materials

Table 1 provides the chemical compositions of the materials used in this study, with all compositions presented as a weight percentage measured by an Inductively Coupled Plasma Optical Emission Spectrometer (ICP-OES) (ICP-5000, Focused Photonics, Hangzhou, China). The material used in this study was prepared as a 150 kg ingot melted under a vacuum and then forged into a 200 mm × 80 mm × 80 mm billet. These billets were then rolled into 15 mm sheets in six passes after being heated to 1200 °C for 40 min, maintaining a final rolling temperature above 850 °C, and then air-cooled to an ambient temperature.

### 2.2. Phase-Transition Temperature and Heating Process

**Figure 1:**
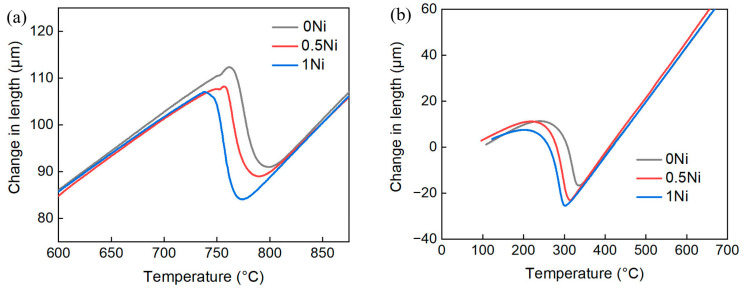
The dilatometric curve of experimental steel with different nickel contents, (**a**) *Ac*_1_, *Ac*_3_; (**b**) *M*_s_.

The dilatometer (DIL 805 A/D TA Instruments, Hüllhorst, Germany) was used to measure the *Ac*_1_, *Ac*_3_, *M*_s_, and continuous cooling transformation (CCT) curves for heat treatment. Samples for the dilatometer were Φ4 × 10 mm^3^ in size. To determine the temperatures of *Ac*_1_, *Ac*_3_, and *M*_s_, the sample was gradually heated at 10 °C/s until it reached 500 °C and then to 900 °C at 0.05 °C/s before finally cooling to an ambient temperature of 50 °C/s. The dilatometric curve and phase transition temperatures of experimental steel with varying nickel contents are displayed in Figure 1 and Table 2, respectively. The *M*_s_ temperature of experimental steel reduced significantly with the increasing nickel content. Moreover, as the nickel content increased from 0 wt.% to 1 wt.%, the *M*_s_ temperature reduced from 345 °C to 318 °C, aligning well with the empirical calculation formula included in the relevant literature [19].

*M*_s_ = 492 − 125*x*C(wt.%) − 65.5*x*Mn(wt.%) − 10*x*Cr(wt.%) − 29*x*Ni(wt.%), °C (1)

Nickel can enlarge the γ phase region in the Fe-C phase diagram, and the *Ac*_3_ temperatures of the tested steel exhibited a decreasing trend with an increasing nickel content, which is consistent with the related literature [16,20]. The *Ac*_3_ temperature of the test steel with 0% nickel was 828 °C, while 900 °C was the austenitization temperature.

Samples were austenitized at 900 °C for 5 min and then cooled to an ambient temperature at rates of 2, 5, 10, 20, and 50 °C/s in order to ascertain the CCT curves. The procedure for the heat treatment process involved austenitizing hot-rolled sheets of 0 Ni, 0.5 Ni, and 1 Ni steel, which were austenitized at 900 °C for 15 min and quenched to room temperature, and then were immediately tempered at 210 °C for 45 min and air-cooled to an ambient temperature.

### 2.3. Mechanical Tests

Tensile tests were conducted on cylindrical specimens with the dimensions of Φ5 mm × 25 mm, which were machined along the rolling direction in accordance with the GB/T 228.1-2021 standard [21]. The AG-X 250 kN (Shimadzu machine, Kyoto, Japan) was used to run tests at 2 mm/min under room temperature conditions. Charpy impact tests were conducted on U-notched specimens measuring 10 mm × 10 mm × 55 mm, with 2 mm of the U-notch prepared following GB/T 229-2016 [22]. Subsequently, these specimens were tested by the Charpy pendulum impact test machine (ZBC2452-B SANS, Shenzhen, China) under different temperatures. The microhardness of the experimental steel plates was measured (Fm-820 Future-tech, Kawasaki, Japan), and the average value was calculated from six data points with a load of 1000 g applied.

### 2.4. Microstructure Observation

To determine the prior austenite grain size (PAGS), samples were polished and etched using a saturated aqueous picric acid solution with 3–4 drops of hydrochloric acid. The martensite packets and blocks were characterized by employing electron back-scattered diffraction (EBSD) and scanning electron microscope (SEM) techniques. The surface of the samples was analyzed using SEM (Ultra 55 Zeiss, Baden-Wurttemberg, Germany) after Nital etching. To eliminate the deformed surface, X-ray diffraction (XRD) and EBSD samples were electropolished using a mixture of 10 vol% perchloric acid and 90 vol% alcohol. The Electromet4 Buehler was utilized for electrolytic polishing at 22 V for a duration of 30 s. EBSD maps were acquired at 20 kV, with the specimen tilted at 70° and a step size of 0.05 μm using a Gimini300 (Zeiss Baden-Wurttemberg, Germany) microscope equipped with an OXFORD system. Subsequently, EBSD data were processed through the AZteccrystal 2.1 software. XRD measurements were conducted at a scanning speed of 2°/min within the range of 40° to 100° using a D/max2400 (Rigaku machine, Tokyo, Japan) apparatus with a Cu-Kα radiation source (λ = 1.5406 Å).

Transmission electron microscopy (TEM) analysis was carried out on a Tecnai G2 F20 (FEI microscope, Hillsboro, OR, USA). TEM thin foils were prepared via fine grinding to a thickness of 50 μm. The discs were then prepared by punching this material into 3 mm circles before they were perforated via electropolishing using a solution of 8% perchloric acid and 92% alcohol at a temperature of −20 °C and with a TenuPol-5 (Struers machine, Copenhagen, Denmark). Image J 1.46r software was used to measure the average volume fraction and diameter of the precipitates from the TEM micrographs.

## 3. Results

### 3.1. CCT Curves of Steel

**Figure 2:**
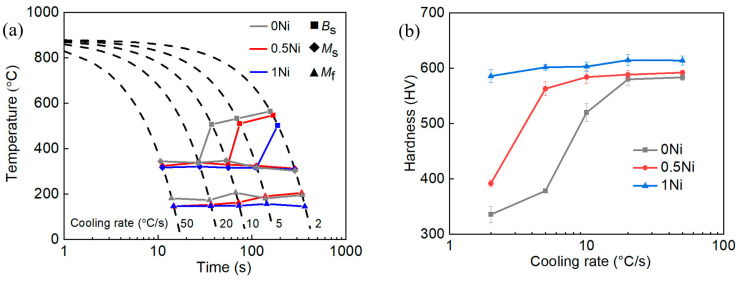
(**a**) The continuous cooling transformation (CCT) curves of experimental steel; (**b**) Hardness of experimental steel at different cooling rates.

Figure 2a shows the continuous cooling transformation (CCT) curves of the spring steel. The 0 Ni steel curves are displayed in gray, while the curves of 0.5 Ni steel and 1 Ni steel are depicted in red and blue, respectively. The *B*_s_ temperature of spring steel decreased significantly as the nickel content increased from 0 wt.% to 1 wt.%. This is because adding nickel reduces the chemical driving force for the nucleation and growth of the bainite transformation, hindering its kinetics, which enhances steel hardenability [20]. According to the hardness distribution diagram in Figure 2b, when the cooling rate reached 20 °C/s, the hardness of all three types of steel exceeded 550 Hv. The critical cooling rates to obtain a complete martensitic microstructure varied between 20 °C/s, 10 °C/s, and 5 °C/s for 0 Ni steel, 0.5 Ni steel, and 1 Ni steel, respectively. The thickness specification of the leaf spring exhibited a positive correlation with its hardenability. Currently, the development of leaf springs is moving towards variable cross-section single leaf springs, which require spring steel with higher hardenability. Adding the element Ni can improve the hardenability of the material, thereby expanding the potential applications of the new generation of high-strength leaf spring steels [2].

### 3.2. Microstructural Characterization

**Figure 3:**
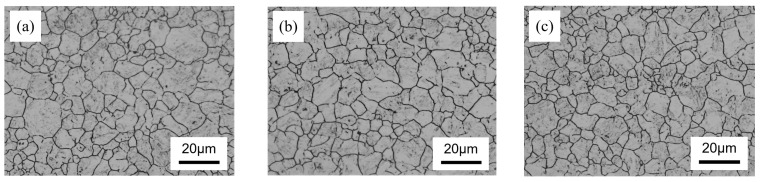
Prior austenite grain of experimental steel with different nickel contents, (**a**) 0 Ni, (**b**) 0.5 Ni, and (**c**) 1 Ni.

Figure 3 shows the typical morphologies of PAG in experimental steel with varying nickel content. The grain size is determined using the linear intercept method followed by the ASTM E 112 standard [23], which involves counting the intersections of grain boundaries with a test line and calculating the average length of these intercepts. This measurement is used to determine the size of the prior austenite grains by analyzing at least eight random images per sample using six lines. The grain sizes of the steel with different nickel contents exhibited uniformity after austenitization at 900 °C. Moreover, the grain size showed a slight decrease with an increase in the nickel content, and the grain sizes for 0 Ni steel, 0.5 Ni steel, and 1 Ni steel were measured as 7.4 ± 0.7 μm, 7.2 ± 0.5 μm, and 6.9 ± 0.6 μm, respectively.

**Figure 4:**
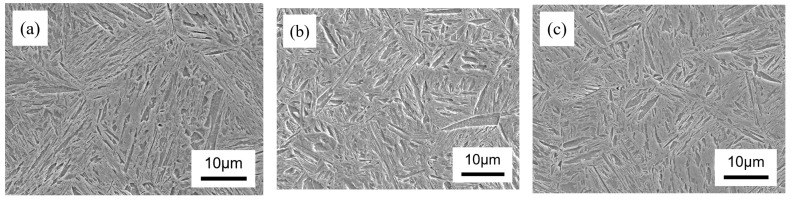
SEM images of experimental steel, (**a**) 0 Ni, (**b**) 0.5 Ni, and (**c**) 1 Ni.

Figure 4 shows the microstructure of experimental steel with different nickel contents after heat treatment. The heat treatment resulted in a tempered martensitic microstructure, showing an insignificant variation amongst the experimental types of steel that contain different nickel contents.

**Figure 5:**
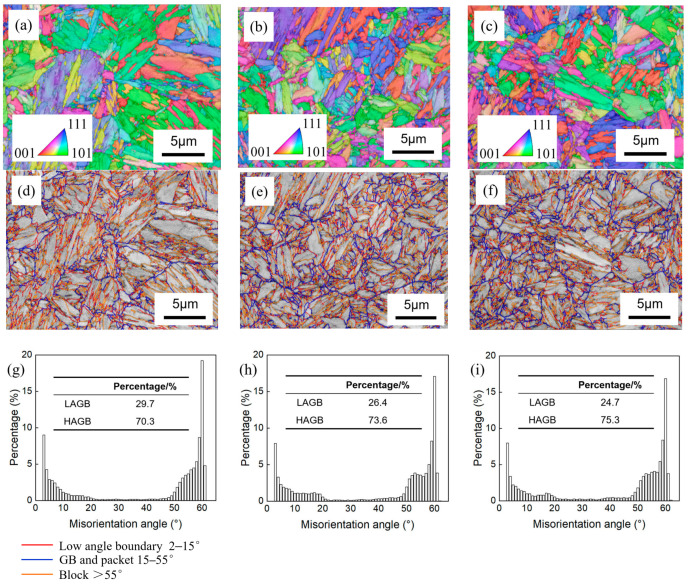
(**a**–**c**) Inverse pole figure (IPF) maps, (**d**–**f**) misorientation and boundary mapping on the hierarchical martensitic microstructure, and (**g**–**i**) distribution of correlated misorientations of experimental steel: (**a**,**d**,**g**) 0 Ni, (**b**,**e**,**h**) 0.5 Ni, and (**c**,**f**,**i**) 1 Ni.

EBSD boundary maps and inverse pole figure (IPF) maps of 0 Ni, 0.5 Ni, and 1 Ni steel are shown in Figure 5. The EBSD orientation mapping technique effectively showcases the typical hierarchical structure of martensite. The low-angle grain boundaries (LAGB) are defined by misorientation angles ranging from 2° to 15°, indicated by red lines, while high-angle grain boundaries (HAGBs) are defined by misorientation angles above 15°. To visualize the martensitic substructure, the HAGBs were classified into the following two categories: grain boundaries and packet boundaries. They are denoted by blue lines with misorientation angles less than 55°, and block boundaries are indicated by orange lines with angles greater than 55° [24]. Figure 5g–i illustrates the misorientation distributions of experimental steel. It is observed that the proportion of HAGBs increases considerably as the nickel content decreases. This study reveals that the percentages of HAGBs in 0 Ni, 0.5 Ni, and 1 Ni steel are approximately 70.3%, 73.6%, and 75.3%, respectively. The addition of nickel refined the PAGS and reduced the *M*_s_ temperature, as shown in Table 2, refining the martensitic substructure and increasing the proportion of HAGBs in experimental steel.

The packet size (PS) and block size (BS) of experimental steel were analyzed using SEM (Figure 4) and EBSD (Figure 5), respectively; the corresponding results are collated in Table 3. The average values of PS and BS were determined by measuring a total of 300 packets and 300 blocks, respectively. The PAGS, PS, and BS of experimental steel with different nickel contents showed a consistent trend: the increase in the nickel content led to a refinement in both the grain size and martensitic substructure in experimental steel, as shown in Table 3. In 0 Ni steel, the PS was higher than in the 0.5 Ni and 1 Ni steel. The PS decreased from 2.5 μm in 0 Ni steel to 2.2 μm in 0.5 Ni steel and 2.1 μm in 1 Ni steel due to the decrease in PAGS. The BS of 0.5 Ni and 1 Ni steel were about 0.9 μm and 0.8 μm, indicating similar values, while the BS decreased rapidly from 1.16 μm in 0 Ni steel to 0.84 μm in 1 Ni steel.

**Figure 6:**
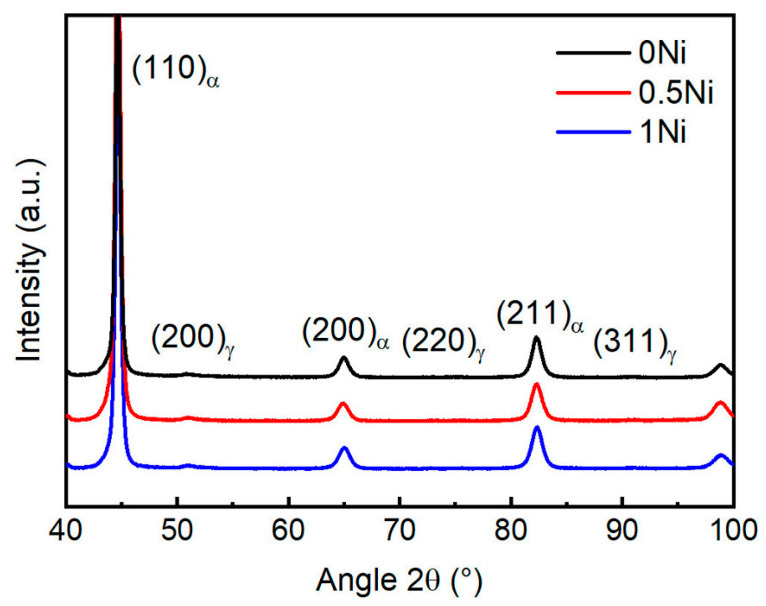
XRD patterns of experimental steel.

The XRD patterns of experimental steel with different nickel contents are shown in Figure 6 (0 Ni/black; 0.5 Ni/red; 1 Ni/blue). Notably, the diffraction peak of the retained austenite was absent from the observed XRD pattern, suggesting that the presence of retained austenite could be disregarded. The Williamson-Hall (W-H) method has long been used to characterize dislocation density [25]. The dislocation density of martensitic steel calculated by the W-H method could always be overestimated due to the strong strain anisotropy of martensitic steel [26]. The modified Williamson-Hall (MWH) method takes into account the effect of strain anisotropy by defining a scaling parameter $\bar{C}$, called the average contrast factor of dislocations [27]. The modified Williamson-Hall (MWH) method was used to calculate the dislocation in medium-carbon martensitic steel [28].

(2) $$∆K≅\frac{0.9}{D}+bM\sqrt{\frac{\pi }{2}\rho }(K{\bar{C}}^{1/2})$$

where Δ*K* is the full width at half-maximum (FWHM). *D* is the crystallite size, which can be obtained using *D* = *kλ*/*βcosθ*. *M* is the dislocations distribution parameter, and *M* = 2 [29]. *B* is the Burgers vector, and *b* = 0.248 nm [28]. *K* is the magnitude of the diffraction vector, which can be obtained using *K* = 2*sinθ*/*λ*. *θ* and *λ,* which are the diffraction angle and the wavelength of the X-rays. $\bar{C}$ is the average contrast factor of dislocations [27,28]. The value of *ρ* obtained via XRD results was estimated to be about 4.20 × 10^15^ m^−2^, 4.71 × 10^15^ m^−2^, and 4.84 × 10^15^ m^−2^ for 0 Ni, 0.5 Ni, and 1 Ni steel.

**Figure 7:**
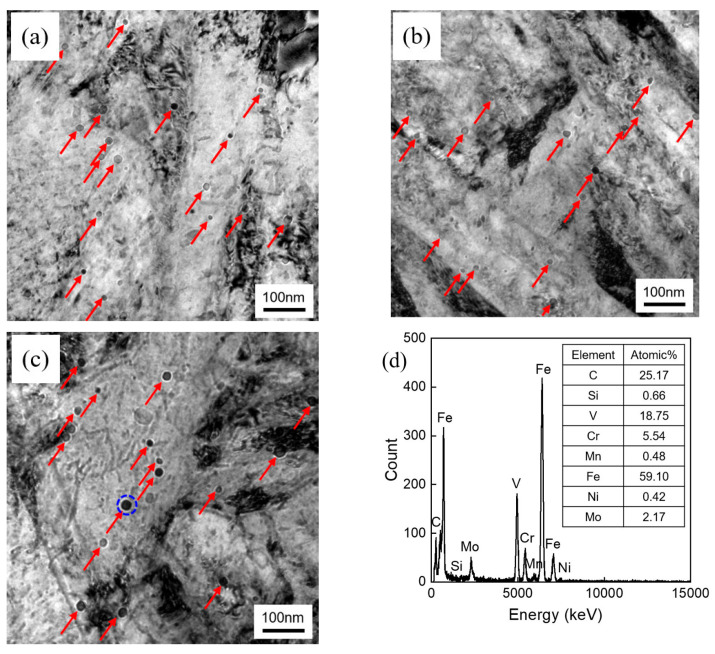
TEM images of experimental steel, (**a**) 0 Ni, (**b**) 0.5 Ni, and (**c**) 1 Ni, and (**d**) the EDS of the precipitates with the blue circle in (**c**).

The TEM results reveal that the microstructure of the experimental steel predominantly consisted of lath martensite, with a significant dispersion of VC particles within the lath structure (Figure 7). The VC carbides in Figure 7 has been marked with red arrows. According to the energy dispersive spectroscopy (EDS) analysis, as shown in Figure 7d, the spherical carbides were identified as VC carbides. The average diameter and volume fraction of VC were measured using Image-Pro Plus 6.0 software, and the results are shown in Table 4, revealing insignificant disparities for the 0 Ni, 0.5 Ni, and 1 Ni steel.

### 3.3. Tensile Properties

**Figure 8:**
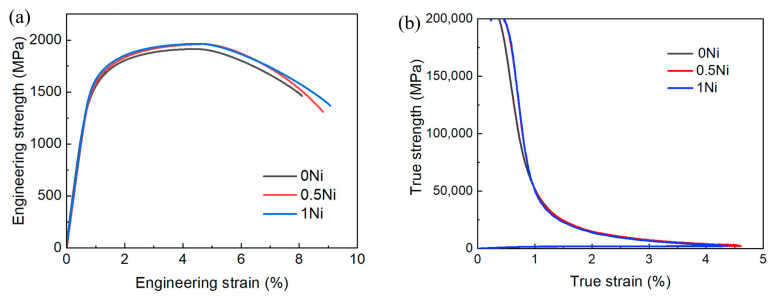
(**a**) The engineering stress–strain relationship of experimental steel; (**b**) Variation in the work hardening rate with the true strain.

The results of the mechanical tensile tests are shown in Figure 8 for 0 Ni, 0.5 Ni, and 1 Ni steel, colored black, red, and blue, respectively, and the values for yield strength (YS), ultimate tensile strength (UTS), and elongation (EL) are summarized in Table 5. As the nickel content increased from 0 wt.% to 1 wt.%, the YS increased significantly from 1503 MPa to 1613 MPa. The 0.5 Ni steel had a similar ultimate tensile strength (~1960 MPa) and total elongation (8.8%) as the 1 Ni steel but a slightly lower yield strength. The ultimate tensile strength (~1910 MPa) and elongation (~8.1%) of the 0 Ni steel were also lower than those of the 0.5 Ni and 1 Ni steel, while its work-hardening behavior was significantly reduced compared to that of 0.5 Ni/1 Ni steel (Figure 8b).

## 4. Discussion

### 4.1. Strengthening Mechanisms

The effect of adding nickel on the individual strengthening mechanisms was further clarified by analyzing their contributions to the yield strength. The yield strength is combined with several strengthening factors according to the following equation [30].

(3) $$\sigma_{y}=\sigma_{0}+\sigma_{ss}+\sigma_{g}+\sigma_{p}+\sigma_{\rho },\;MPa$$

where *σ*_0_ is the lattice friction stress of pure Fe, *σ*_0_ = 54 MPa [31], *σ_ss_* is the solid solution hardening stress, *σ_g_* is the grain boundary hardening stress, *σ_ρ_* is the dislocation hardening stress, and *σ*_p_ is the precipitation hardening stress.

The solid solution hardening stress (*σ_ss_*) based on the alloy composition can be calculated using the following equation [14,32].

(4) $$\sigma_{ss}=4570xC(wt.\%)+4570xN(wt.\%)+37xMn(wt.\%)+83xSi(wt.\%)+11xMo(wt.\%)+\;0xNi(wt.\%)+3xV(wt.\%)-30xCr(wt.\%),\;\mathrm{MPa}$$

Interstitial carbon atoms are effective for solution strengthening. Wilde et al. [33] provided the observations of the interstitial carbon in martensitic steel by three-dimensional atomic-scale mapping. The results indicate that most of the carbon elements tend to segregate at the grain boundaries and dislocation lines rather than occupying interstitial positions. In martensitic steel, carbon atoms exist in a solid solution with an interstitial content of about 0.02 wt.% [34]. C = 0.02 wt.% has been widely used in the calculation of strengthening mechanisms, especially in the calculation of 2000 Mpa martensitic steel [35]. Ni as a replacement atom has no significant effect on the amount of solid solution of elemental C in martensitic steels. Therefore, in this study, the solid solution strengthening effect of the carbon element was calculated on the basis of 0.02 wt.% for 0 Ni, 0.5 Ni, and 1 Ni steels. The concentrations of substitutional atoms are consistent in 0 Ni, 0.5 Ni, and 1 Ni steel, resulting in a uniform value of 158 MPa due to the nickel showing no solid solution strengthening [14].

Grain boundary hardening stress can be calculated using the Hall–Petch equation [36].

(5) $$\sigma_{g}=k_{y}d^{-1/2},\;MPa$$

where the coefficient *k_y_* is taken to be 0.21 MPa m^1/2^ [37], and *d* is the effective grain size in μm.

The Hall–Petch strengthening mechanism results from the accumulation of dislocations. It is widely accepted that high-angle boundaries (θ > 15°) can impede the dislocation slip [36]. The HAGBs in the structure, including the prior austenite grain, packet, and block boundaries, have a misorientation of θ > 15°. The block width is significantly smaller than the packet size, suggesting that the HAGBs in the structure are mainly influenced by the block boundaries [22,38]. Therefore, the block size in this article was selected as the effective grain size. The concentrations of grain boundary hardening stress were calculated and are listed in Table 6.

Precipitation hardening stress (*σ*_p_) can be estimated using the following equation [14].

(6) $$\sigma_{p}=10.8\frac{f_{V}^{1/2}}{X}ln\frac{X}{6.125\times 10^{-4}},\;MPa$$

where *f_V_* is the volume fraction of the second precipitate particle, and *X* is the average size of the second precipitate particle in μm. The average diameter and volume fraction of VC is measured in Figure 7. The precipitation hardening stress was calculated and is given in Table 6.

The dislocation hardening stress (*σ_ρ_*) can be estimated using the following equation [32].

(7) $$\sigma_{\rho }=\alpha MGb\sqrt{\rho },\;MPa$$

where *α* is the constant with a value of 0.25 [39], *M* is the Taylor orientation factor, *M* = 2.75 [40], *G* is the shear modulus of Fe, *G* = 78 Gpa [40], *b* is the Burgers vector, *b* = 0.248 nm [40], and *ρ* is the dislocation density calculated using the XRD results. The dislocation hardening stress of experimental steel was calculated and is listed in Table 6.

**Figure 9:**
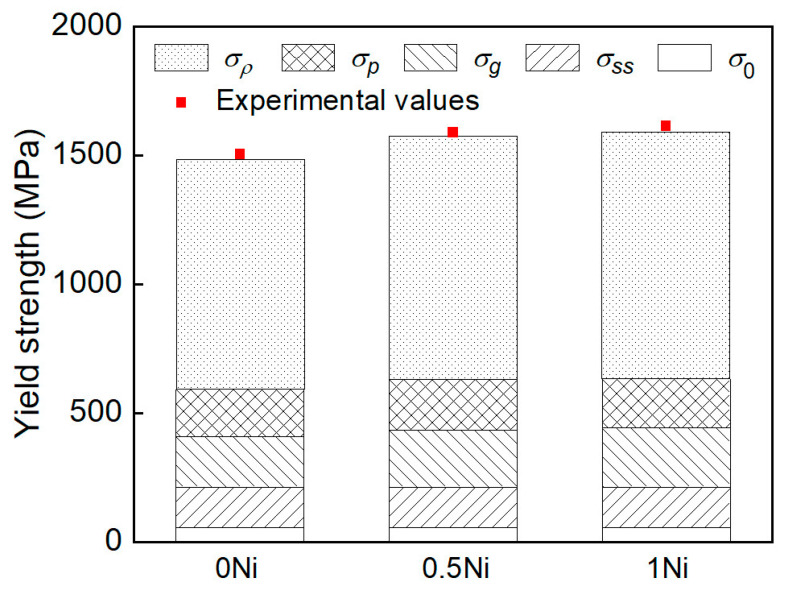
Comparison between the experimental and calculated values and the contributions of strengthening in experimental steel.

The contribution of individual strengthening factors to the yield hardening stress was calculated using Equations (3)–(7) and given in Table 6 and Figure 9, showing good agreement with the experimental values, with a deviation less than +25 MPa calculated by $\sigma_{exp}$−$\sigma_{cal}$. The deviation may be attributed to the statistical errors in determining the average diameter and volume fraction of precipitation, as well as the dislocation density. As the nickel content increased from 0 wt.% to 1 wt.%, the grain boundary hardening stress increased from 195 MPa to 229 MPa while the dislocation hardening stress increased from 895 MPa to 961 MPa. This is mainly due to the increase in Ni concentration, which refines the sub-microstructure of martensite and inhibits the precipitation of cementite during the tempering process, effectively increasing the yield strength of the material. The results indicate that adding nickel plays an important role in the yield strength through a synergistic combination of grain refinement strengthening and dislocation strengthening, while lattice friction stress of pure Fe, precipitation hardening stress, and solid solution hardening stress are close for 0 Ni, 0.5 Ni, and 1 Ni steel.

### 4.2. Impact Properties

**Figure 10:**
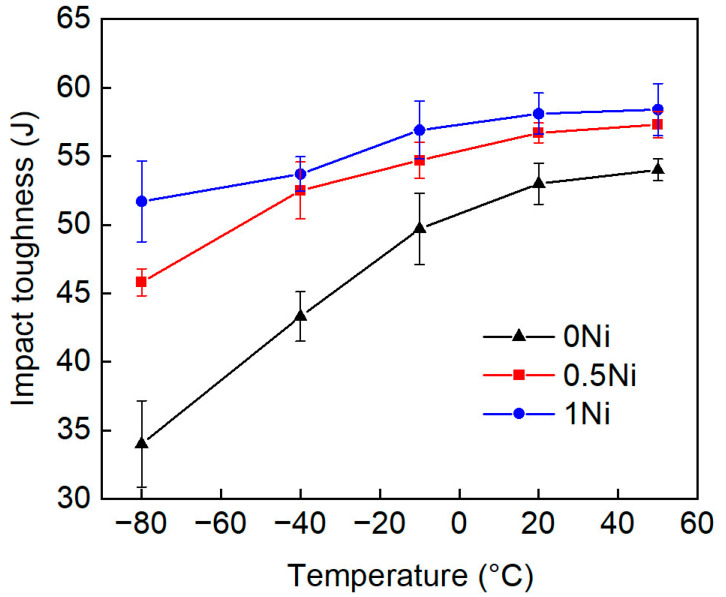
Temperature evolution of the absorbed energy measured for the 0 Ni, 0.5 Ni, and 1 Ni steel in the range of −80 to 50 °C.

Figure 10 (0 Ni/black; 0.5 Ni/red; 1 Ni/blue) and Table 7 show the Charpy-absorbed energy of 0 Ni, 0.5 Ni, and 1 Ni steel when tested at different temperatures ranging from −80 °C to 50 °C. The test temperature was above the brittle transition temperature of the steel. Therefore, the ductile-to-brittle transition temperature (DBTT) cannot be determined. The impact toughness of 0 Ni, 0.5 Ni, and 1 Ni steel showed a similarity (above 50 J) when the test temperature was above −10 °C. The increase in the nickel content resulted in a slight increase in the impact toughness, which could be attributed to the refinement of the package size (Table 3). In the −40 °C impact test, the impact toughness of the FG microstructure (43 J) was much lower than that of the other two types of steel (~53 J). When tested at −80 °C, the impact toughness of the 0 Ni, 0.5 Ni, and 1 Ni steel was approximately 34 J, 46 J, and 52 J, respectively. The inclusion of packet boundaries and block boundaries within the HAGBs significantly hindered the propagation of fractures, thereby effectively reducing the DBTT of martensitic steel [41,42,43]. Increasing the nickel content from 0 wt.% to 1 wt.% resulted in a decrease in the packet size from 2.5 μm to 2.1 μm and the block size from 1.2 μm to 0.8 μm (Table 3), accompanied by an increase in the proportion of HAGBs from 70% to 75% (Figure 5). This shifts the brittle temperature zone toward lower temperatures and effectively improves the cryogenic impact toughness.

## 5. Conclusions

The effects of nickel addition on the microstructure and mechanical properties of medium-carbon spring steel have been investigated. The results are summarized as follows:

(1) The addition of nickel enlarges the γ phase region, improves the hardenability, and reduces the *M*_s_ temperature of medium-carbon spring steel.
(2) The packet size (PS) and block size (BS) of the experimental steel decrease with increasing Ni content, while the percentage of high angle grain boundaries (HAGBs) increases significantly.
(3) The 0.5 Ni steel exhibits a good combination of ultimate tensile strength (1958 MPa) and yield strength (1613 MPa) with a total elongation of 8.8%, with a 54 J absorbed energy testing at −40 °C. The yield strength of medium-carbon spring steel increases by approximately 100 MPa due to a synergistic combination of grain refinement strengthening and dislocation hardening, resulting from an increase in the nickel content from 0 wt.% to 1 wt.%.
(4) The cryogenic impact toughness of the medium-carbon spring steel shows an increasing trend with an increasing nickel content due to the refinement of the sub-microstructure of martensite (including packet and block) and the increase in the proportion of HAGBs, which shifts the brittle temperature zone to lower temperatures.

## Figures and Tables

**Table 1:** The chemical compositions of the investigated steel (wt.%).

Steel	C	Mn + Si + Mo	Cr	Ni	V	P	S	Fe
0 Ni	0.35	1.44	0.98	0	0.21	0.005	0.005	Bal.
0.5 Ni	0.35	1.34	1.03	0.52	0.22	0.005	0.005	Bal.
1 Ni	0.34	1.38	1.04	1.05	0.21	0.005	0.005	Bal.

**Table 2:** *Ac*_1_, *Ac*_3_, and *M*_s_ temperatures of experimental steel with different nickel contents.

Steel	*Ac*_1_ (°C)	*Ac*_3_ (°C)	*M*_s_ (°C)
0 Ni	741	828	345
0.5 Ni	745	818	326
1 Ni	739	796	318

**Table 3:** PAGS, packet size (PS), and block size (BS) of experimental steel.

Steel	PAGS (μm)	PS (μm)	BS (μm)
0 Ni	7.4 ± 0.7	2.5 ± 0.4	1.16 ± 0.48
0.5 Ni	7.2 ± 0.5	2.2 ± 0.2	0.91 ± 0.29
1 Ni	6.9 ± 0.6	2.1 ± 0.2	0.84 ± 0.34

**Table 4:** The average volume fraction and diameter of VC.

Steel	Average Volume Fraction (%)	Average Diameter (nm)
0 Ni	0.18 ± 0.03	10.11 ± 0.11
0.5 Ni	0.21 ± 0.05	10.20 ± 0.09
1 Ni	0.19 ± 0.03	10.05 ± 0.15

**Table 5:** Mechanical properties of experimental steel with different nickel contents.

Steel	YS (MPa)	UTS (MPa)	EL (%)
0 Ni	1503 ± 6.4	1912 ± 5.8	8.1 ± 0.2
0.5 Ni	1588 ± 5.6	1963 ± 6.3	8.8 ± 0.3
1 Ni	1613 ± 5.2	1958 ± 3.8	8.8 ± 0.3

**Table 6:** Estimated contributions of individual strengthening factors to yield strength and experimental results for the experimental steel (MPa).

Steels	$\sigma_{0}$	$\sigma_{ss}$	$\sigma_{g}$	$\sigma_{p}$	$\sigma_{\rho }$	$\sigma_{cal}$	$\sigma_{exp}$	Deviation
0 Ni	54	158	195	184	895	1486	1503	17
0.5 Ni	54	158	220	198	947	1577	1588	11
1 Ni	54	158	229	190	961	1592	1613	21

**Table 7:** Absorbed energy measured for the 0 Ni, 0.5 Ni, and 1 Ni steel.

Steel	−80 °C	−40 °C	−10 °C	20 °C	50 °C
0 Ni	34.2 ± 3.2	43.3 ± 1.8	50.1 ± 2.6	53.2 ± 1.5	54.1 ± 0.8
0.5 Ni	45.8 ± 0.9	52.5 ± 2.1	54.7 ± 1.3	56.9 ± 0.7	57.3 ± 0.9
1 Ni	51.7 ± 2.9	53.7 ± 1.3	56.9 ± 2.1	58.1 ± 1.5	58.4 ± 1.9

## Data Availability

Data are contained within the article.
